# Optimization of ivacaftor-loaded solid lipid nanoparticles for solubility enhancement

**DOI:** 10.3389/fphar.2025.1619481

**Published:** 2025-08-20

**Authors:** Akshay Parihar, Bhupendra G. Prajapati, Himanshu Paliwal

**Affiliations:** ^1^ Faculty of Pharmacy, Ganpat University, Mehsana, Gujarat, India; ^2^ Faculty of Pharmaceutical Sciences, The ICFAI University, Himachal Pradesh, India; ^3^ Department of Pharmaceutics, Parul Institute of Pharmacy, Faculty of Pharmacy, Parul University, Waghodia, Vadodara, Gujarat, India; ^4^ Centre for Research Impact and Outcome, Chitkara College of Pharmacy, Chitkara University, Rajpura, Punjab, India; ^5^ Department of Industrial Pharmacy, Faculty of Pharmacy, Silpakorn University, Nakhon Pathom, Thailand; ^6^ Marwadi University Research Center, Faculty of Pharmacy, Marwadi University, Rajkot, Gujarat, India

**Keywords:** CFTR modulator, solid-lipid nanoparticles, solubility enhancement, *in vitro* dissolution, release kinetics

## Abstract

**Background:**

Cystic fibrosis (CF) is a systemic disease which primarily affects pulmonary system, but also extends to different important organs to cause multitude of associated diseases, leading to rise in rate of morbidity and mortality. The present investigation is focused on the development and optimization of SLN (Solid Lipid Nanoparticles) formulation of IVF (Ivacaftor) for effective treatment of cystic fibrosis.

**Methods:**

IVF-SLN was formulated with the help of homogenization and ultrasonication methods by incorporating Labrasol as liquid lipid, Cetyl palmitate as solid lipid and Polysorbate 20 as the surfactant. The independent variables such as the amount of Lipid (X_1_) and amount of surfactant (X_2_) were studied for their effect on dependent variables namely entrapment efficiency and particle size.

**Results:**

The final formulation of IVF-SLN showed a narrow range in size distribution with the particle size of 150.23 ± 1.59 nm, PDI of 0.256 ± 0.014 and entrapment efficiency of 90.54 ± 1.32%. IVF incorporation into the imperfect crystal lattice was confirmed with the help of a DSC (Differential Scanning Calorimetry) study. The *in vitro* dissolution study showed improved release pattern from the optimized formulation and release profile followed first-order model indicating a sustained release pattern from lipid matrix.

**Conclusion:**

This delivery system presented development of stable nanoparticle formulation exhibiting sustained release pattern, which may contribute to therapeutic outcomes in comparison with drug alone. The outcomes of the research highlighted its potential as an efficient therapeutic strategy for CF management.

## 1 Introduction

Cystic fibrosis (CF) follows an autosomal recessive behavior of inheritance which can be characterized by the presence of mutations in the CFTR (cystic fibrosis transmembrane regulator) gene. CF is recognized as a systemic disease that can extend beyond the involvement of the pulmonary system including various vital organs causing a multitude of associated diseases (Jiang et al., 2024; Lou et al., 2024). Mortality and morbidity are greatly affected when the pulmonary pathway is disturbed by CF (Kim et al., 2023). The situation is exaggerated by a series of events which include excessive mucus production, bacterial infections, obstruction of the respiratory airway, inflammation, and termination of lung diseases (Ong and Ramsey, 2023).

The formulations targeting the pulmonary system are growing exponentially and it has become a welcoming approach for the treatment of cystic fibrosis and other chronic obstructive pulmonary disease. Significant biomedical attention has been garnered by this field. This pulmonary route helps in avoiding the first-pass metabolism and reduces the adverse effect with enhanced drug aggregation at the site of action (Anjum et al., 2023). Administering the drug through pulmonary route also helps in reducing the dose frequency and increasing patient compliance. The significant gaps between the current therapeutic regimen and nanotherapeutics can be filled by improving the intracellular uptake, deposition kinetics and drug release in a controlled manner at the action site. Advancements in nanotechnology have helped in rapidly developing nanoparticles for medical purposes (Taylor-Cousar et al., 2023). Pulmonary diseases can be efficiently treated with the help of specially designed nanoparticles or nanocapsules. They help in covering the overlapping concepts by understanding exhaled breath chemistry (Das et al., 2011).

The advancement in the formulation of SLN over the years has helped in addressing various aspects of SLNs, method of preparation, their composition and stability properties (Paliwal et al., 2023). The general properties such as morphology, particle size, stability and release behaviour for the solid lipid nanoparticles remain the same whether some modifications on the surface have been performed (Almawash, 2023). The loading capacity and encapsulation efficiency of the introduced bioactive may be affected if they are soluble in the lipids and there may be any interaction between them. Despite the versatility of the SLN, they also exhibit some limitations like low loading capacity of drugs, and polymeric transition on storage causing drug explosion (Tang et al., 2023). Invasive treatment of the chronic diseases with biomolecules is limited due to their instability in biological system. Pulmonary delivery of nanoparticles poses a promising alternative for their delivery in local as well as systemic administration. Due to the ability of SLNs to carry hydrophobic and hydrophilic agents, exploration is still remaining in the areas of lung infections, cystic fibrosis and lung cancer (Alwani et al., 2024). Liposomes offer better site targeting and drug release but their major disadvantage is intrinsic complexity which makes them inadequate for nano-systems (Wang et al., 2024). Therefore, a standardized regulatory approach is required for optimizing the acceptance of methodologies so that clinical translation can be accelerated (Giordani et al., 2023).

Drug release is inversely related to its partition coefficient and to achieve controlled drug release, it is required to increase the surface area and homogenous dissemination of drug in lipid matrix. Sensitive pharmaceuticals can also be protected from degradation by formulating SLN as a carrier. This facilitates regulation and targeting of the drug molecule (Phalak et al., 2024; Yuan et al., 2025). With the improvement of pharmaceutical technology, QbD (Quality by Design) has also become synonymous with it. QbD principles and approaches have been embraced by pharmaceutical companies as well for the development of the product and understanding of the process (Camacho Vieira et al., 2024). The therapeutic action of the SLN is improved due to their enhancement in their sustained release profile. Implying QbD in the formulation of SLNs has shown improved entrapment efficiency and cellular uptake (Chin et al., 2025). Since formulation of nanoparticles is a complex process, Implying QbD techniques has improved the product reliability and its quality (Birla et al., 2025).

The focus of present work is to integrate design approach for solubility enhancement of Ivacaftor by SLN formulation. The process parameters and formulation parameters were optimized using the statistical principles for obtaining desirable physiochemical properties. The optimized IVF loaded SLNs were able to exhibit enhanced *in vitro* drug release suggesting its impending improved bioavailability. This study emphasizes the design and development of nanocarriers rationally to overcome the challenging molecules like IVF.

## 2 Materials and methods

### 2.1 Materials

IVF (Ivacaftor) was obtained from Alembic Pharmaceuticals Limited Vadodara as a gift sample. Polysorbate 20, SLS (Sodium Lauryl Sulphate), PEG 40 (Polyethylene Glycol 40), Soy lecithin, Polysorbate 60, and Polysorbate 80 were used as Surfactant. They were obtained as a gift sample from Gattefossé. Oleic acid, Isopropyl myristate, Capryol 90, Labrasol and Labrafac were used as Liquid lipids and were obtained as gift samples from Gattefossé. Miglyol 812, Compritol, Trimyristin, Triolein, Palmitic acid, Cetyl Palmitate, and Stearic acid were used as liquid lipids and were obtained as gift samples from Coral Pharma Limited.

### 2.2 Measurement of IVF solubility in lipids

The solid lipids hampered the equilibrium solubility study; therefore, an unconventional method was required to measure the solubility of the drug in solid lipids. 10 mg of Ivacaftor was accurately weighed and placed in a bottle of glass. An excess amount of lipid was weighed and added to the glass bottle. It was heated to 80° C with continuous stirring. Additional lipid was added in separate portions until a clear solution was formed. A record was maintained to calculate the amount of lipid added until a clear solution was formed (Das et al., 2011).

The solubility study for the liquid lipids and surfactants was performed by adding an excess amount of drug to glass vials in triplicate containing each of the excipients. The glass vials were placed on a vortex mixture for 15 min and placed in an incubator at 37° C during the period of solubility study. Vials were vortexed periodically so that the drug could be kept suspended. Samples were taken at 24, 48 and 72 h so that it can reach the equilibrium. The vials were subjected to centrifugation at 37° C, 2,800 rpm for 30 min two to three drops of the supernatant which is equivalent to 100–150 µL were transferred in a tared container and diluted with methanol for quantification (Shah et al., 2015). The measurement of absorbance was determined by the actual concentration of IVF at the wavelength of 384.3 nm with the help of a UV-Vis (Ultraviolet visible) spectrophotometer (UV-1800, Shimadzu, Japan). The component compatibility with IVF was determined with the help of FT-IR (Fourier Transform Infrared spectroscopy), and they were found to be compatible with IVF.

### 2.3 Formulation of IVF-loaded SLN

**FIGURE 1:**
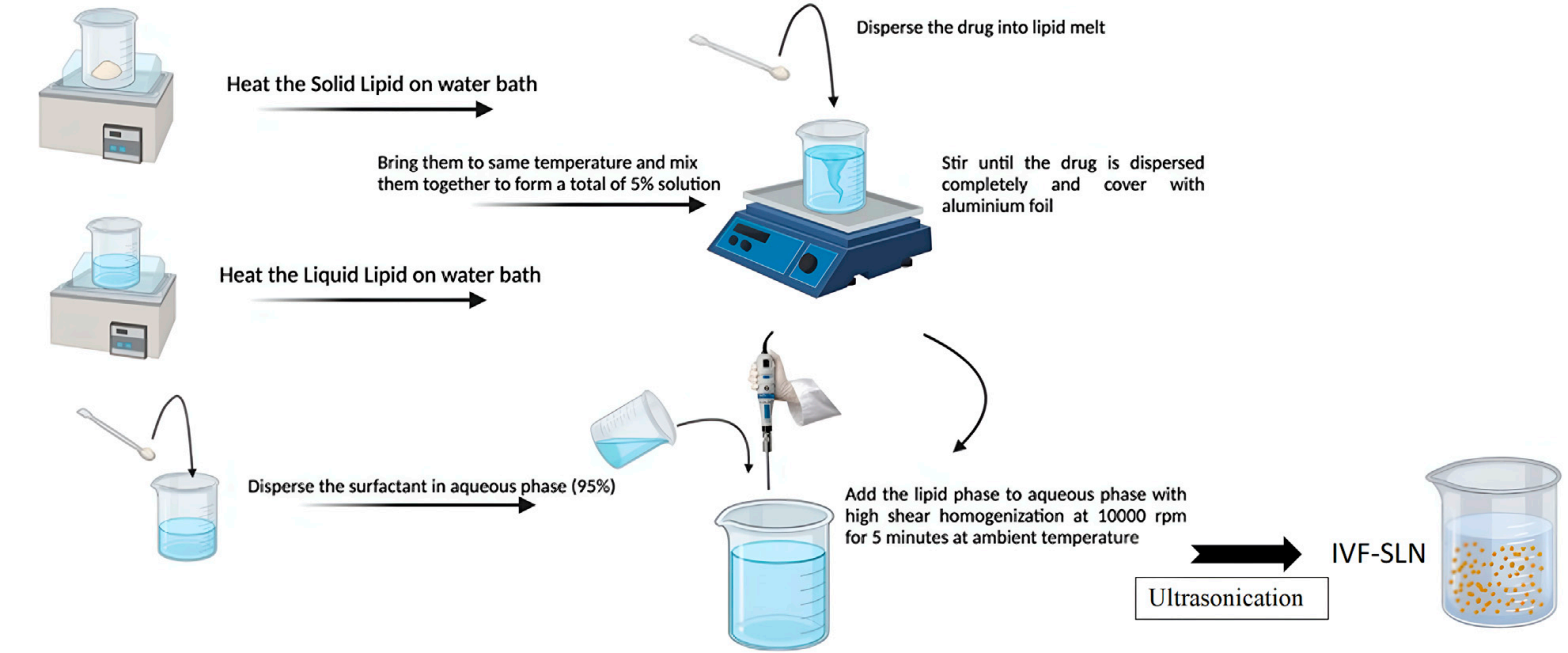
Schematic diagram showing method of preparation of IVF-SLN.

IVF-SLNs were prepared by emulsification-ultrasonication method. The solid lipid was heated in a water bath at 80° C and a liquid lipid was heated separately in a water bath and brought to the same temperature. The drug was dispersed into lipid melt and stirred as shown in Figure 1. The liquid lipid and solid lipid were mixed at the same temperature to disperse the drug completely. Surfactant was dispersed in the aqueous phase forming a 95% solution. The lipid phase was mixed with the aqueous phase by bringing them to the same temperature. This mixture was subjected to high-speed homogenization at 10,000 rpm for 5 min at ambient temperature. The formation of primary emulsion was followed by ultrasonication with the help of a probe sonicator keeping the constant temperature. The formed lipidic dispersion was allowed to cool at room temperature for 15 min to form the IVF-SLN (Mehnert and Mäder, 2012).

### 2.4 Experimental design

The independent variables and their identification were done with the help preliminary investigations for the determination of their minimal and maximal values. The central composite design (CCD) was used for the optimization of IVF-SLN to study the effect of two independent variables viz.*,* amount of lipid (X_1_) and amount of surfactant (X_2_) were determined at two levels of entrapment efficiency and particle size (Table 1). The face-centered CCD with alpha = 1 and single center point were employed in the design. Labrasol and cetyl palmitate were selected as liquid-lipid and solid-lipid respectively (Paliwal et al., 2022).

**TABLE 1:** Factors and Responses for central composite design for optimization of IVF-SLN formulation.

Independent variables	Levels		
	Low (−1)	Medium (0)	High (+1)
X_1_: Amount of lipid (mg)	40	100	160
X_2_: Amount of surfactant (mg)	10	20	30
Dependent Variables			
Y_1_: % Entrapment efficiency (%)			
Y_2_: Particle size (nm)			

Factorial design was analyzed using Design Expert software (Version 13.0.5, Stat-Ease Inc., Minneapolis, USA) which helped in deriving the polynomial equation. The coefficient of the polynomial equation has a magnitude that has either a negative sign for the antagonistic effect or a positive sign for the synergistic effect. The statistical parameters like multiple correlation coefficient, coefficient of variation, predicted residual sum of a square and adjusted multiple correlation coefficient were analyzed to select the best-fitting experimental model (Das et al., 2011).

### 2.5 Data optimization and validation of model

The design space was created to study the effect of independent variables on dependent variables. Therefore, a CCD was used to develop the design space and study the response on the process parameters to ensure the quality of IVF-SLN (Suresh et al., 2007). The optimization was based on enhancing encapsulation efficiency and lowering the particle size. Checkpoints were analyzed to establish the reliability of the developed model (Parihar and Prajapati, 2023).

### 2.6 Characterization of IVF-SLN

The Particle size, PDI (Polydispersity Index) and Zeta potential were analyzed using a Zetasizer (NanoZS, Malvern Instruments, UK). The IVF-SLN was diluted with double distilled water 10 times. PDI and Particle size measurements were performed by taking out 1 mL of diluted formulation into cuvettes of polystyrene and disposable folded capillary cells at 25°C respectively. The centrifugation method was used to determine the %EE (Entrapment Efficiency) of IVF-SLN. The sample was centrifuged at 10,000 rpm for 20 min to obtain a pellet of lipid nanoparticles at room temperature. The supernatant was diluted by methanol and free drug content was analyzed using UV spectroscopy at 384.3 nm (Rahman et al., 2010). The %EE was calculated by following equation: (Total amount of IVF-Amount of free IVF)/Total amount of IVF, pH (Thermo scientific, USA) of IVF-SLN was analyzed by taking the formulation in a beaker in a volume of 10 mL. A calibrated digital pH meter was used to measure the pH at room temperature. DSC (DSC-60, Shimadzu) analysis of IVF, Labrasol, Physical mixture, blank SLN and IVF-SLN were performed. The samples were lyophilized before DSC analysis.

### 2.7 Morphological characterization

SLNs were observed under scanning electron microscopy for morphological studies. For SEM (30 kV EVO-18 Carl Zeiss Scanning Electron Microscope) analysis, SLN was loaded with Drugs using a two-way tape coated with gold in a vacuum sputter coater.

### 2.8 *In vitro* drug release study of IVF-SLN

A further attempt was made to develop the *in vitro* drug release method of SLN by dialysis method using a bottle rotating apparatus. The dosage form was placed in a glass bottle with dissolution medium SGF (Simulated Gastric Fluid) pH 1.2 and SIF (Simulated Intestinal Fluid) pH 6.8 separately in pre-warmed condition at 37°C to evaluate the effect of pH on the formulation’s release profile. These conditions simulate the gastric and intestinal environment. The formulation was to be administered through oral route and it will have to encounter different pH, so it was essential to assess the release profile of the formulation in acidic and near-neutral environments. The bottle is sealed and place in rotating apparatus to avoid evaporation. The rotation was set on a constant speed of 25 rpm. The dissolution medium was withdrawn at predetermined intervals of 5, 10, 15, 20, 30, and 45 min and analyzed for the drug content. The medium was replaced with fresh medium to maintain the sink condition. The simulated gastric fluid of pH 3.4 was used as a dissolution medium to check the impact on drug release (Harivardhan Reddy et al., 2006). The *in vitro* release data of pure IVF, optimized IVF-SLN, and marketed formulations were fitted to different mathematical models, such as Zero-order, First-order, Higuchi, Hixson-Crowell, and Korsmeyer-Peppas to estimate the release kinetics. The model fitting was evaluated on the basis of correlation coefficient (R^2^), and release constants were generated from the respective model equations (Altas et al., 2025).

## 3 Results

### 3.1 Solubility of IVF in solid lipids

**FIGURE 2:**
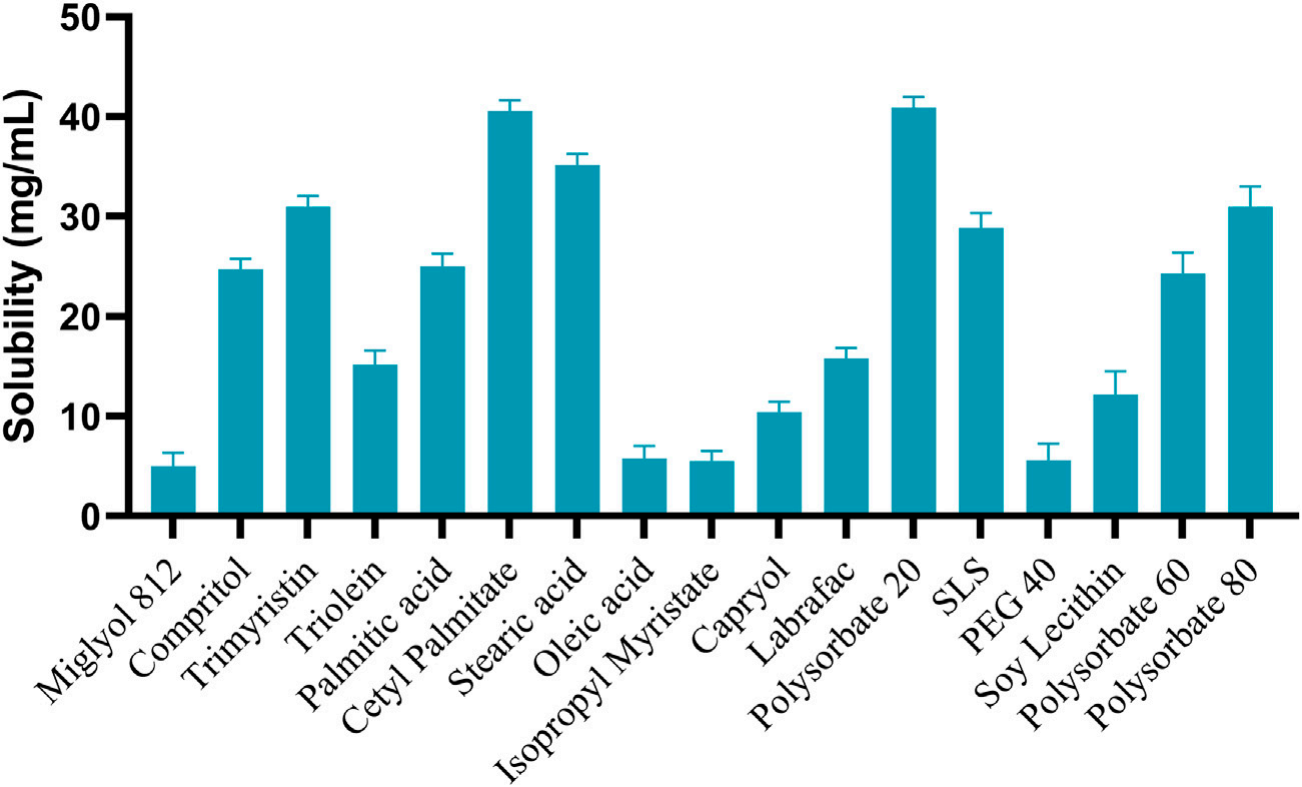
Drug solubility study in surfactant, Solid-lipid, and liquid-lipids (Mean ± SD, n = 3).

In the formulation of SLN, entrapment of the drug in solid lipid becomes a limiting factor. The entrapment in turn affects the %EE. As per the data shown in Figure 2, several different solid lipids, liquid lipids and surfactants were analyzed to perform the solubility study. Among them, Cetyl palmitate, Labrasol and Polysorbate 20 showed the highest solubility as solid-lipid, liquid-lipid, and surfactant respectively. Therefore, they were taken for further formulation (Iazzi et al., 2023).

### 3.2 Preliminary screening of lipids, surfactant and high-speed homogenizer (HSH)

Three batches of SLN were prepared using the solid lipid in which Cetyl palmitate in D: L (drug: lipid) (1:2) showed the lowest size. Hence, cetyl palmitate was selected as an optimized lipid, further 1% w/w of polysorbate 20 was taken as a stabilizer. The SLN which has been stabilized with the help of surfactant shows lower particle size and improved stability. When we increased the HSH rpm the size and PDI were increased gradually and % entrapment efficiency was decreased.

### 3.3 Experimental design

A factorial design was used to determine the influence of two factors, namely, the amount of lipid (X_1_) and surfactant (X_2_) and the result of their interaction on the responses over particle size and entrapment efficiency. The factorial design matrix was prepared. The results helped identify how factors and their interaction significantly influenced responses. A greater slope indicates greater influence over the variables in the system. The significant statistical level of each factor and their effect was determined as shown in Table 2.

**TABLE 2:** Optimization of processing parameters (Values are indicated as Mean ± SD).

Formulation code	Factors		Responses	
	Amount of lipid (mg)	Amount of surfactant (mg)	Entrapment efficiency (%) (Y_1_)	Particle size (nm) (Y_2_)
IVF-SLN1	40	10	55.86 ± 2.12	163.24 ± 2.15
IVF-SLN2	40	20	60.82 ± 2.08	166.84 ± 3.06
IVF-SLN3	100	20	67.95 ± 1.46	169.65 ± 2.96
IVF-SLN4	160	20	75.62 ± 1.62	178.28 ± 2.47
IVF-SLN5	100	10	61.82 ± 2.11	165.90 ± 2.29
IVF-SLN6	40	30	60.83 ± 1.41	164.44 ± 2.73
IVF-SLN7	160	10	72.28 ± 1.04	175.86 ± 3.01
IVF-SLN8	100	30	70.45 ± 1.68	168.62 ± 2.03
IVF-SLN9	160	30	78.32 ± 2.06	182.66 ± 1.88

#### 3.3.1 Effect of independent variables on % entrapment efficiency (Y_1_)

A linear model was observed to be best fitted for assessing the influence of lipid, and surfactant on %EE. The model was found to be statistically significant as F-value of 131.47 and p-value of <0.0001 was estimated by ANOVA, suggesting that the selected variables have considerable impact over the response. The fitted regression equation for %EE is as follow:

$$Y_{1}=67.11+8.12X_{1}+3.27X_{2}$$

**FIGURE 3:**
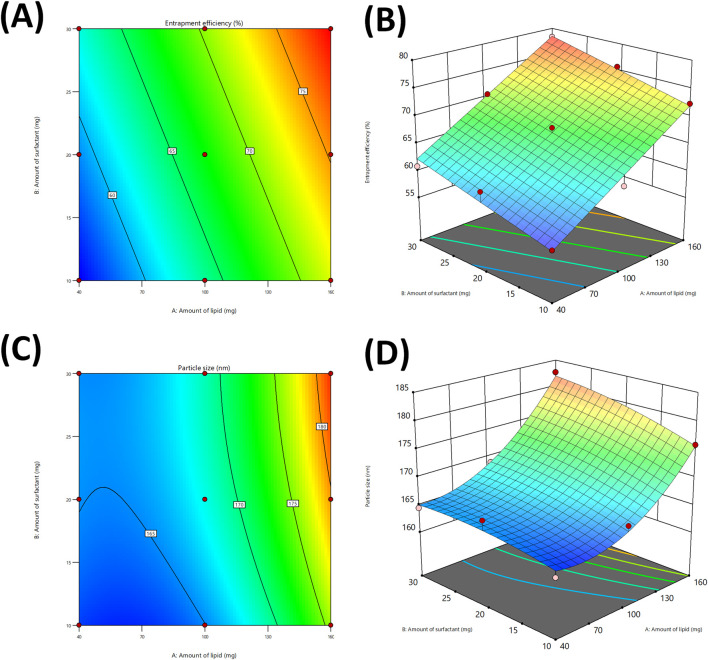
Contour plots for studying the effect of independent variables on %entrapment efficiency **(A)**, and Particle size **(C)**. 3D response plot generated for %entrapment efficiency **(B)**, and Particle size **(D)**.

The contour and 3D response surface plots (Figures 3A,B) indicated that an increment in lipid concentration (X_1_) led to significant increase in %EE. This can be ascribed to the presence of extensive lipid matrix, enabling more drug molecules to be entrapped in the nanoparticles (Puri et al., 2009), Furthermore, the surfactant concentration (X_2_) indicated a mild positive effect on %EE, possibly owing to improved emulsification, which further enhanced the dispersion of drug in the lipid phase (Mittal et al., 2024).

#### 3.3.2 Effect of independent variables on particle size (Y_2_)

The response surface analysis for particle size (Y_2_) followed a significant quadratic model, with a p-value of 0.0082 and model F-value of 32.24, indicating a reliable model fit. The derived regression equation is:

$$Y_{2}=169.03+7.04X_{1}+1.78X_{2}+1.4X_{12}+3.83{X_{1}}^{2}-1.47{X_{2}}^{2}$$

The ANOVA results and corresponding plots (Figure 3C,D) indicated that the lipid concentration had the most substantial effect on the particle size. The increment in lipid concentration resulted in a considerable increase in particle size, possible due to improved viscosity and aggregation of lipid droplets during homogenization (Pan et al., 2024). Although, the surfactant concentration had lesser impact but still played role in reduction of particle size, likely owing to its contribution in stabilization of the lipid droplets and avoiding coalescence (Sakellari et al., 2022). The modest interaction effect indicates that the appropriate regulation of both factors is vital to ensure optimal size range. The slight negative coefficients for squared surfactant concentration indicated an inverse relationship with particle size.

### 3.4 Validation of model

**FIGURE 4:**
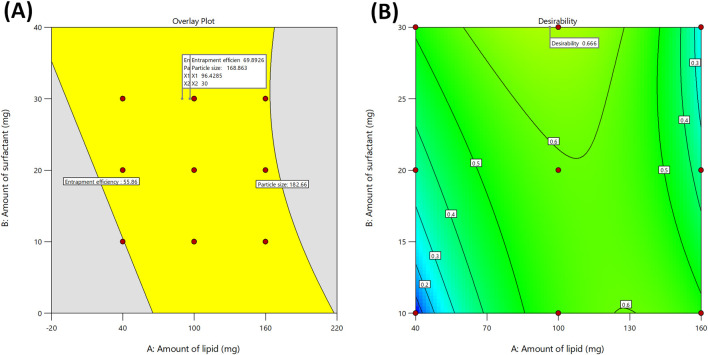
**(A)** Overlay plot for optimization of the Ivacaftor-loaded SLN, and **(B)** Contour plot representing overall desirability function of optimized formulation.

All formulations were subjected to experimental trials. With the help of desirability function, the independent variables were optimized with their respective responses. The responses Y_1_ and Y_2_ were converted into separate desirable scales respectively. The desirability and overlay plot (Figure 4) of the DoE contributed optimum values of both factors (Amount of lipid, and amount of surfactant), from which the final formulation was prepared. The composition of final optimized formulation was Labrafac-10%, Cetyl Palmitate 16.75%, and Polysorbate 10.5%. The optimized IVF-SLN formulation (IVF-SLN8) was developed for checkpoint analysis and characterized for entrapment efficiency, and particle size, showing response variables of Y_1_ = 70.54%, and Y_2_ = 150.23 nm (Table 3). The predicted and observed values were in agreement with each other as indicated by desirability value of 0.666 (Figure 4B).

**TABLE 3:** Point prediction.

Analysis	Predicted Mean	Observed	Std Dev	Desirability
Entrapment efficiency (%)	67.1056	70.54	1.32	0.666
Particle size (nm)	167.812	150.23	1.49	

### 3.5 Characterization of optimized IVF-SLN

The outcomes of characterization of formulation are indicated in Table 4. IVF-SLN formulations were characterized for zeta potential and the values of zeta potential ranged from −21.5 ± 1.2 mV for IVF-SLN1 to −27.5 ± 1.3 mV for IVF-SLN9. The functional groups on the lipid and surfactant components of the formulation were ionized which attributed to the negative surface charge. Zeta potential values of higher magnitude suggested that there was a stronger electrostatic repulsion between the particles causing an improved stability of the colloidal system. IVF-SLN7, IVF-SLN8, IVF-SLN9 were having higher lipid content, and they showed a more negative values of zeta potential from −25.7 to −27.7 mV. This was an indicative of enhancement in physical stability, and it also reduce the risk of aggregation. The values of zeta potential were in the range of ±20 to ±30 mV which is considered acceptable for nano-based formulations.

**TABLE 4:** Outcomes of Zeta Potential, PDI, and pH of different IVF-SLN formulations (Values are indicated as Mean ± SD).

Formulation Code	Zeta potential (mV)	PDI	pH
IVF-SLN1	−21.5 ± 1.2	0.316 ± 0.014	6.51 ± 0.07
IVF-SLN2	−23.1 ± 1.0	0.279 ± 0.011	6.56 ± 0.06
IVF-SLN3	−24.6 ± 1.2	0.241 ± 0.009	6.47 ± 0.04
IVF-SLN4	−25.3 ± 1.1	0.228 ± 0.013	6.42 ± 0.05
IVF-SLN5	−22.7 ± 0.9	0.262 ± 0.010	6.49 ± 0.04
IVF-SLN6	−23.6 ± 1.1	0.286 ± 0.012	6.55 ± 0.08
IVF-SLN7	−26.0 ± 1.2	0.233 ± 0.006	6.44 ± 0.05
IVF-SLN8	−25.7 ± 1.5	0.209 ± 0.008	6.48 ± 0.03
IVF-SLN9	−27.5 ± 1.3	0.191 ± 0.007	6.41 ± 0.06

The value of PDI for all the formulations were in the range from 0.191 ± 0.007 for IVF-SLN9 to 0.316 ± 0.0014 for IVF-SLN1 indicating uniform distribution of particle size. PDI value below 0.3 indicates that the monodisperse system has a homogenous distribution of particles which is a desirable property of a reproducible drug delivery system. The lowest PDI values of 0.209 and 0.191 were shown by IVF-SLN8 and IVF-SLN9 respectively, it is associated with the optimized level of surfactant and balanced lipid matrix formulating a stabilized particle system and improving the emulsification. Whereas IVF-SLN1 exhibited highest value of PDI reflecting a poor lipid-to-surfactant ratio and hence causing a broader distribution of particle size.

The pH of formulations for IVF-SLN9 to IVF-SLN2 were in the range from 6.41 ± 0.06 to 6.56 ± 0.06 respectively. The pH was in near neutral range which is a critical parameter for biological tissue compatibility. It was concluded that all the formulations were well withing the acceptable range therefore, no additional adjustment of pH was required.

**FIGURE 5:**
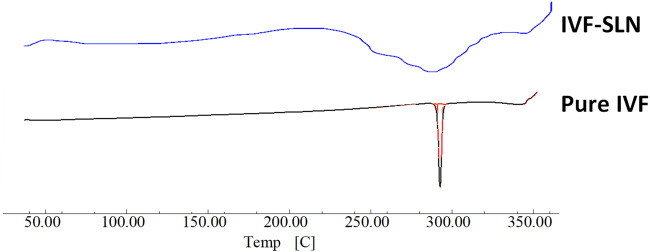
Comparative DSC curve of pure IVF and Optimized IVF-SLN formulations.

**FIGURE 6:**
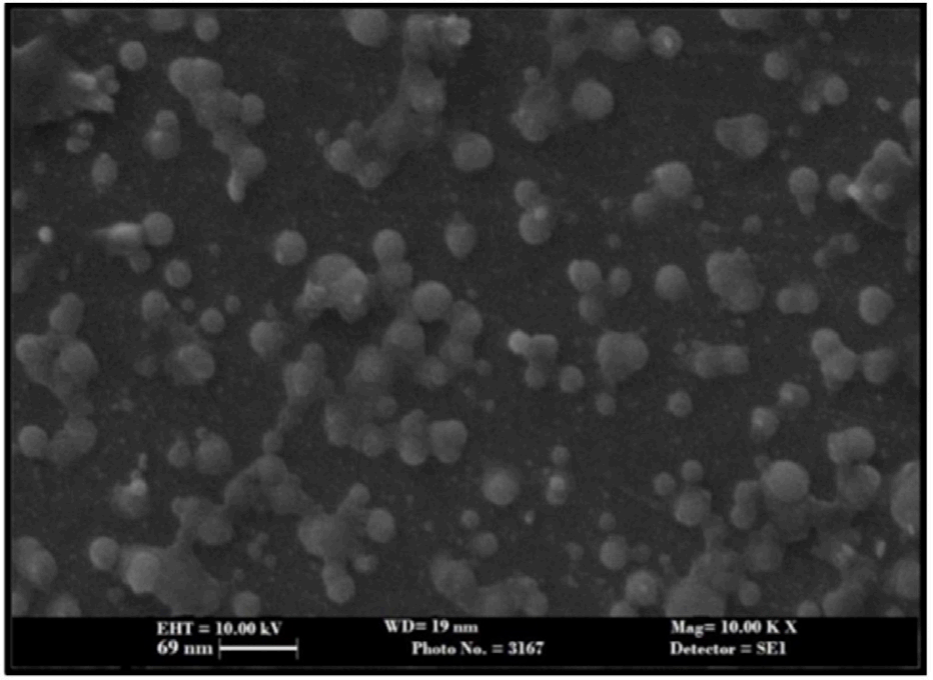
SEM image of Optimized IVF-SLN formulations (Scale bar = 69 nm).

As shown in Figure 5 the DSC curve of pure ivacaftor and IVF-SLN8 formulation. The IVF curve shows a sharp peak indicating crystallinity. In IVF-SLN, the enthalpy of cetyl palmitate was lowered. This was indicative of crystalline to amorphous form conversion of SLN formulations as it was incorporated in the melted lipid matrix. The morphological evaluation of IVF-SLN8 with the help of SEM analysis showed spherical-shaped SLN as indicated Figure 6 which were in the size range of 70–190 nm which was further agreed with the size distribution analysis based on the dynamic light scattering principle.

### 3.6 *In vitro* drug release study of IVF-SLN

**FIGURE 7:**
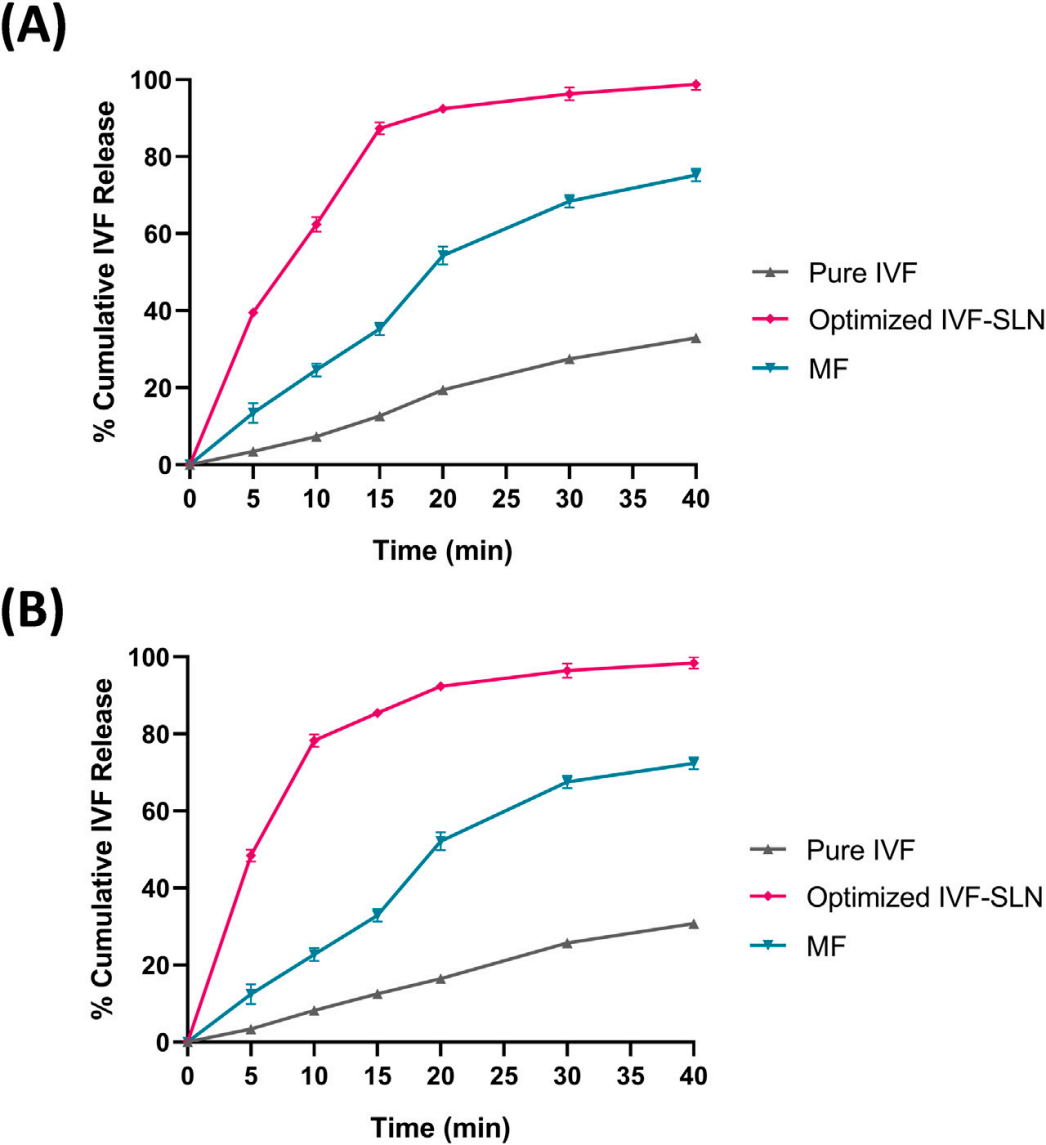
*In-vitro* drug release for the optimized formulations in **(A)** SGF pH 1.2 & **(B)** SIF pH 6.8 and comparison with marketed formulation (MF) and pure IVF.

The *in vitro* drug release profiles of the optimized IVF-SLN, pure IVF and the marketed formulation were evaluated in SIF pH 6.8 and SGF pH 1.2 containing 1% methanol. The drug release of IVF-SLN8 was improved significantly in comparison to the marketed drug and the pure drug (Figures 7A, B). The IVF-SLN8 exhibited a rapid initial drug release of 65% within 10 min and 98.78% drug release was achieved within 40 min in SGF pH 1.2 (Figure 7A). A comparable pattern of drug release was in SIF pH 6.8 for IVF-SLN8 and achieved a maximum release of 98.4% within 40 min. An initial burst followed by sustained release was observed due to drug diffusion from the surface of SLN and gradually the lipid matrix was eroded. Whereas pure IVF exhibited a slow and limited release of 40% by 40 min in SGF pH 1.2 as well as SIF pH 6.8. The marketed formulation was able to delivery intermediate release behaviour of 70% within 40 min. The improved particle size and surface area of the nanoparticles lead to a superior performance of IVF-SLN8. The presence of surfactant improved drug solubility and diffusion (Inês Teixeira et al., 2023).

The release kinetics showed that the pure IVF followed first-order kinetics (R^2^ = 0.991 in SGF; 0.995 in SIF), indicating concentration-dependent release (Table 5). Further, the optimized IVF-SLN8 formulation also followed first-order (R^2^ = 0.982 in SGF; 0.979 in SIF), suggesting a controlled, sustained release pattern possibly controlled by diffusion and erosion of lipid matrix. Apart from first-order model, the Higuchi model also showed high R^2^ values for formulations (0.922 for SGF and 0.893 for SIF), indicating that the release mechanism is affected by diffusion of drug from lipid matrix. The Korsmeyer-Peppas model showed n values more than 1 (1.221 in SGF; 1.201 in SIF) for IVF-SLN8, suggesting super case-II transport, which supports the mechanism that polymer relaxation and erosion along with diffusion. Contrarily, the marketed formulation showed a best fit with first-order and zero-order models, indicating a uniform but rapid release in comparison with IVF-SLN8. Also, pure IVF demonstrated rapid release kinetics with low K_1_ values but with superior fit first-order kinetics, indicating rapid solubilization.

**TABLE 5:** Fitting of *in vitro* release data to different mathematical models.

Kinetic models	Parameters	Pure IVF		IVF-SLN8		Marketed formulation	
		SGF	SIF	SGF	SIF	SGF	SIF
Zero Order	R^2^	0.987	0.993	0.718	0.642	0.955	0.953
	K_0_	0.873	0.800	2.219	2.031	1.961	1.917
First-order	R^2^	0.991	0.995	0.982	0.979	0.985	0.976
	K_1_	0.011	0.010	0.111	0.102	0.037	0.035
Higuchi	R^2^	0.898	0.910	0.922	0.893	0.941	0.930
	K_H_	5.556	5.111	16.791	15.987	12.993	12.644
Hixson-crowell	R^2^	0.990	0.995	0.913	0.886	0.980	0.972
	K_HC_	0.015	0.014	0.089	0.082	0.046	0.044
Korsmeyer-peppas	R^2^	0.988	0.993	0.848	0.811	0.968	0.973
	K_KP_	0.868	0.902	2.218	2.463	1.373	1.326
	n	0.990	0.962	1.221	1.201	1.179	1.174

## 4 Conclusion

In our present work, we used the experimental design approach to develop hydrophilic IVF loaded Solid lipid nanoparticles. IVF-loaded SLN was prepared using homogenization and ultrasonication. We studied the individual effect of critical processing parameters on critical quality attributes using CCD so that the desired quality of the product can be achieved. We obtained the optimum formula for the formulation with the help of an overlay plot. SLN having an entrapment efficiency greater than 65% is indicative of a higher incorporation of IVF. IVF-SLN is lipidic which helps in enhanced drug diffusion in comparison to crystalline drugs. The results were further validated with the help of DSC and FTIR data which showed that IVF had lost its crystalline characteristic. We can conclude that QbD can be used to successfully develop an SLN-based carrier system with better quality attributes.

## Data Availability

The original contributions presented in the study are included in the article/supplementary material, further inquiries can be directed to the corresponding author.
